# Systemic Responses of Multidrug-Resistant *Pseudomonas aeruginosa* and *Acinetobacter baumannii* Following Exposure to the Antimicrobial Peptide Cathelicidin-BF Imply Multiple Intracellular Targets

**DOI:** 10.3389/fcimb.2017.00466

**Published:** 2017-11-07

**Authors:** Cunbao Liu, Bin Shan, Jialong Qi, Yanbing Ma

**Affiliations:** ^1^Laboratory of Molecular Immunology, Institute of Medical Biology, Chinese Academy of Medical Sciences and Peking Union Medical College, Kunming, China; ^2^Department of Clinical Lab, The First Affiliated Hospital of Kunming Medical University, Kunming, China

**Keywords:** antimicrobial peptide pressure, cathelicidin-BF, intracellular targets, multidrug-resistant, *Pseudomonas aeruginosa*, *Acinetobacter baumannii*, comparative proteomics

## Abstract

Cathelicidin-BF, derived from the banded krait (*Bungarus fasciatus*), is a typically cationic, amphiphilic and α-helical antimicrobial peptide (AMP) with 30 amino acids that exerts powerful effects on multidrug-resistant (MDR) clinical isolates, including *Pseudomonas aeruginosa, Acinetobacter baumannii*, and *Klebsiella pneumoniae*, but whether it targets plasma membranes or intracellular targets to kill bacteria is still controversial. In the present study, we demonstrated that the disruption of bacterial membranes with high concentrations of cathelicidin-BF was the cause of bacterial death, as with conventional antibiotics at high concentrations. At lower concentrations, cathelicidin-BF did not cause bacterial plasma membrane disruption, but it was able to cross the membrane and aggregate at the nucleoid regions. Functional proteins of the transcription processes of *P. aeruginosa* and *A. baumannii* were affected by sublethal doses of cathelicidin-BF, as demonstrated by comparative proteomics using isobaric tags for relative and absolute quantification and subsequent gene ontology (GO) analysis. Analysis using the Kyoto Encyclopedia of Genes and Genomes showed that cathelicidin-BF mainly interferes with metabolic pathways related to amino acid synthesis, metabolism of cofactors and vitamins, metabolism of purine and energy supply, and other processes. Although specific targets of cathelicidin-BF must still be validated, our study offers strong evidence that cathelicidin-BF may act upon intracellular targets to kill superbugs, which may be helpful for further efforts to discover novel antibiotics to fight against them.

## Introduction

The bacterial pathogens *Pseudomonas aeruginosa* and *Acinetobacter baumannii* are the top two causes of pneumonia acquired in intensive care units (ICUs) and ventilator-associated pneumonia (VAP), with mortality rates of 37.4 and 34.5%, respectively (Zhang et al., 2014). Emerging resistance to colistin and tigecycline, two of the few choices for last-resort treatment of *P. aeruginosa* and *A. baumannii*, may make this situation worse (Cai et al., 2012; Deng et al., 2014; Potron et al., 2015; Lee et al., 2016). Some antimicrobial peptides (AMPs) have shown excellent effects on these drug-resistant pathogens *in vitro* but are not able to be administered systemically due to their shortcomings, such as hemolytic activity or poor stability *in vivo* (Ramamoorthy et al., 2006; Zetterberg et al., 2011; Vila-Farres et al., 2012; Li et al., 2014; Liu et al., 2015). Though efforts have been made, mainly based on structural design, to overcome these disadvantages, no AMP is clinically available to date (Fjell et al., 2011; Andres, 2012). Instead of taking the original AMPs as structural templates, the understanding of their unique mechanisms of action, especially those whose targets may be different from those of the antibiotics available at present, may be more helpful, along with the development of advanced computer-aided drug discovery.

The well-known targets of AMPs are negatively charged prokaryotic cell membranes. Their “selective toxicity” induces transmembrane pores that cause the leakage of intracellular components and finally bacterial death while leaving the electrically neutral membranes of eukaryotic cells untouched (Matsuzaki, 1999). This hypothesis is challenged by the fact that some AMPs kill not only bacteria but also viruses, fungi, protozoa, parasites, and cancer cells, and some AMPs have hemolytic activities (Wang et al., 2016). In recent decades, more and more non-membrane targets of AMPs similar to those of conventional antibiotics have been reported. Examples include cell wall synthesis (mersacidin), DNA (tachyplesin, indolicidin), RNA (buforin II) and important proteins (microcin B17, microcin J25, pyrrhocoricin) (Yonezawa et al., 1992; Brotz et al., 1998; Park et al., 1998; Heddle et al., 2001; Kragol et al., 2001; Mukhopadhyay et al., 2004; Brogden, 2005; Hsu et al., 2005; Parks et al., 2007). Because some clinical isolates have gained resistance to nearly all of the antibiotics available yet some AMPs still work, in particular through non-membrane targets, elucidation of their unique modes of action is highly anticipated.

Cathelicidin-BF, derived from the banded krait (*Bungarus fasciatus*), is a typically cationic, amphiphilic and α-helical AMP with 30 amino acids that exerts powerful effects on multidrug-resistant (MDR) clinical isolates, including *P. aeruginosa, A. baumannii* and *Klebsiella pneumoniae*, but its mechanism of action is still controversial (Wang et al., 2008; Zhou et al., 2011; Hao et al., 2013; Liu et al., 2015; Azim et al., 2016). We have noticed that while some reports have tried to explain its mechanism using the membrane rupture thesis, the concentrations of AMP used to support such claims are always higher than their minimal inhibitory concentration (MIC), which makes the interpretation implausible (Zhou et al., 2011; Gao et al., 2015; Yu et al., 2015). Conversely, at concentrations that result in low toxicity to normal mammalian cells, including erythrocytes, cathelicidin-BF was reported to inhibit cancer cell proliferation, possibly via intracellular targets (Tian et al., 2013; Wang et al., 2013). These clues imply that cathelicidin-BF may act on intracellular targets to kill bacteria.

A systemic view of how bacteria react to antibiotics by comparative proteomics or proteome microarray may reflect pathways with which these antibiotics interfere and is helpful to elucidate undefined mechanisms of novel antibiotics, including AMPs (Kohanski et al., 2010; Hessling et al., 2013; Liu et al., 2014; Elnakady et al., 2016; Ho et al., 2016; Pulido et al., 2016). In the present study, we treated MDR *P. aeruginosa* and *A. baumannii* with sublethal doses of cathelicidin-BF, tested the membrane permeability using the DNA-binding fluorescent dye propidium iodide (PI), checked the localization of fluorescein isothiocyanate (FITC)-tagged AMP with confocal microscopy, and further analyzed the differentially expressed proteins by isobaric tags for relative and absolute quantification (iTRAQ) with standard bioinformatics analyses, such as gene ontology (GO) and use of the Kyoto Encyclopedia of Genes and Genomes (KEGG).

## Materials and methods

### Ethics statement

The animal experimental procedures were approved by the Ethics Committee of Animal Care and Welfare of the Institute of Medical Biology, Chinese Academy of Medical Sciences (CAMS) & Peking Union Medial College (PUMC) (Permit Number: SYXK (dian) 2010-0007), in accordance with the animal ethics guidelines of the Chinese National Health and Medical Research Council (NHMRC) and the Office of Laboratory Animal Management of Yunnan Province, China. All efforts were made to minimize animal suffering.

All participants submitted a signed informed consent form to participate in the study. The protocol complied with the Helsinki Declaration and was approved by the Institutional Review Board of the Institute of Medical Biology, CAMS & PUMC.

### Serum stability of cathelicidin-BF

Female BALB/c mice (6–8 weeks old, 16–18 g) were purchased from Vital River Laboratory Animal Technology Co. Ltd., and raised and maintained in the Central Animal Care Services of our institute under specific pathogen-free (SPF) conditions. Mice were anesthetized, and blood samples were collected by cardiac puncture. After being kept at 37°C for 1 h and then at 4°C overnight, blood samples were centrifuged at 3,000 × g for 15 min, and sera were collected. A portion of the serum was inactivated by boiling in a water bath for 20 min. *Escherichia coli* strain DH5α was grown in Luria-Bertani (LB) medium at 37°C with constant shaking at 220 rpm overnight to reach the middle of the logarithmic growth phase and diluted with LB to 3 × 10^5^ CFU/mL before use. For tests, cathelicidin-BF (purity ≥95%, synthesized by GL Biochem Ltd, Shanghai, China) was dissolved in sterile deionized water and mixed with serum, inactivated serum or sterile deionized water at a volume ratio of 1:4 to achieve a final concentration of 2 mg/mL. After incubation at 37°C, aliquots were taken at each time point, and the MIC for DH5α was taken as the lowest peptide concentration at which no microbial growth was observed visually after 18 h of incubation at 37°C.

### Cytotoxicity assay

Cell viability was measured using cell proliferation kit II (XTT) (Roche). Cells, including A549 (adenocarcinomic human alveolar basal epithelial cells), 293FT (human embryonic kidney cells) and L929 (murine fibroblast cells), were cultured in Dulbecco's minimum essential medium (DMEM) supplemented with 10% fetal bovine serum, 100 U penicillin/mL, and 100 μg streptomycin/mL in a humidified 5% CO_2_ atmosphere at 37°C. After digestion with trypsin, the cells were diluted in serum-free DMEM without phenol red to a final concentration of 2 × 10^5^ cell/mL, seeded in 96 well plates (100 μl/well) and cultured overnight until adhesion. Cathelicidin-BF dissolved in serum-free DMEM without phenol red was added to wells, and the plates were incubated for 24 h as previously described. Subsequent procedures were performed according to the kit. Briefly, XTT labeling reagent (sodium 3′-[1-(phenylaminocarbonyl)-3,4-tetrazolium]-bis-4-methoxy-6-nitro) benzene sulfonic acid hydrate) in Roswell Park Memorial Institute (RPMI) 1640 medium without phenol red was mixed with electron-coupling reagent PMS (N-methyl dibenzopyrazine methyl sulfate) at a volume ratio of 50:1 to make the working solution. Fifty microliters of the XTT labeling mixture was added to each well and incubated at the same conditions for 6 h. Absorbance [A492 nm-A690 nm] stands for the quantification of viable cells.

For the hemolysis assay, blood samples from mice were mixed with Alsever's solution (8 g/L sodium citrate, 0.55 g/L citric acid, 20.5 g/L glucose, 4.2 g/L NaCl, pH 6.1) at a volume ratio of 1:5, centrifuged at 1,000 × g for 10 min, and washed three times with 0.9% saline. Erythrocytes were suspended in 0.9% saline at a volume ratio of 1:50. Cathelicidin-BF dissolved in 0.9% saline was added and incubation continued at 37°C for 30 min. Then, the samples were centrifuged at 1,000 × g for 15 min, the supernatants were diluted four times with 0.9% saline, and the absorbance at 540 nm was measured. Using 1% Triton X-100 (v/v) to determine 100% hemolysis and 0.9% saline as the negative control, the hemolysis rate of cathelicidin-BF is expressed as [(Absorbance _sample_-Absorbance _control_)/(Absorbance_100%_-Absorbance _control_)] × 100.

### Membrane permeabilization assay

One MDR clinical isolate, *P. aeruginosa* 1409, was identified with a Vitek 32 system (bioMerieux, France) and further verified by sequencing of 16s rDNA with universal primers 27f and 1492R. For tests, the bacteria were incubated in LB at a concentration corresponding to an OD_600_ value of 0.5, and then cathelicidin-BF was added to 100 μL of culture to obtain final concentrations of 4 × MIC (32 μg/mL) or ¼ × MIC (2 μg/mL). Levofloxacin (Tokyo Chemicals Industry Co. Ltd.,) with final concentrations of 4 × MIC (64 μg/mL) or ¼ × MIC (4 μg/mL) was used as a control. After 1 h of incubation at 25°C, the culture was centrifuged at 3,000 × g for 5 min and resuspended in PBS (phosphate-buffered saline, 8 g/L NaCl, 0.2 g/L KCl, 1.44 g/L Na_2_HPO_4_, 0.24 g/L KH_2_PO_4_, pH 7.4). Then, PI was added to a final concentration of 10 μg/mL. After 30 min of incubation at 25°C, the cells were washed 3 times with PBS and immediately imaged with a fluorescence microscope (Olympus, Japan) (Yu et al., 2015).

### Localization of cathelicidin-BF in viable bacteria

One MDR clinical isolate, *A. baumannii* 1408, was identified with a Vitek 32 system (bioMerieux, France) and further verified by sequencing 16s rDNA with universal primers 27f and 1492R. For tests, bacterial strains *A. baumannii* 1408 and *P. aeruginosa* 1409 were incubated in LB at a concentration corresponding to an OD_600_ value of 0.5, and then cathelicidin-BF conjugated with FITC at its N terminus (purity ≥95%, synthesized by GL Biochem Ltd, Shanghai, China) was added to a final concentration of ¼×MIC (i.e., 4 μg/mL for *A. baumannii* 1408 and 2 μg/mL for *P. aeruginosa* 1409) and incubated at 25°C for 1 h. The culture was centrifuged at 3,000 × g for 5 min, resuspended in PBS, and incubated at room temperature for 20 min with Hoechst (Sigma) diluted with PBS to a final concentration of 20 μg/mL. Next, the culture was centrifuged at 3,000 × g for 5 min, incubated with SynaptoRed C2 (Tocris Bioscience), diluted with Hank's solution (8 g/L NaCl, 0.4 g/L KCl, 1 g/L glucose, 60 mg/L KH_2_PO_4_, 47.5 mg/L Na_2_HPO_4_, pH 7.2) to a final concentration of 20 μg/mL, and maintained on ice for 1 min. Microscopy was performed with excitation and emission wavelengths, respectively, of 488 nm and 530 nm for FITC, 352 nm and 461 nm for Hoechst, and 515 nm and 640 nm for SynaptoRed C2 (Olympus, Japan) (Wang et al., 2015).

### Bacterial protein preparation

Bacterial strains *A. baumannii* 1408 and *P. aeruginosa* 1409 were grown overnight in LB medium at 37°C with constant shaking at 220 rpm to reach the middle of their logarithmic growth phase. Cathelicidin-BF was added to a final concentration of 1/2 MIC (i.e., 8 μg/mL for *A. baumannii* 1408 and 4 μg/mL for *P. aeruginosa* 1409), and the cultures were incubated at 37°C for 2 h. Samples were collected by centrifugation at 4,000 × g for 5 min at 4°C and washed 3 times with PBS. All samples were homogenized in lysis buffer (4% SDS, 1 mM DTT, 150 mM Tris-HCl, pH 8.0, protease inhibitor). After 5 min incubation in boiling water, the homogenate was sonicated on ice. The crude extract was then incubated in boiling water again and clarified by centrifugation at 16,000 × g at 25°C for 10 min before the supernatants were collected. The protein concentration in the supernatants was determined using the BCA protein assay (Beyotime, China).

### Protein digestion and iTRAQ labeling

Protein digestion was performed based on a filter-aided sample preparation procedure (Wisniewski et al., 2009). The resulting peptide mixtures were labeled with the 4-plex iTRAQ reagent according to the manufacturer's instructions (Applied Biosystems). Briefly, 200 μg of proteins for each sample was incorporated into 30 μl STD buffer (4% SDS, 100 mM DTT, 150 mM Tris-HCl, pH 8.0). The detergent, DTT and other low-molecular-weight components were removed using UA buffer (8 M urea, 150 mM Tris-HCl, pH 8.0) by repeated ultrafiltration (Microcon units, 30 kD). Then, 100 μl of 0.05 M iodoacetamide in UA buffer was added to block reduced cysteine residues, and the samples were incubated in darkness for 20 min. The filters were washed with 100 μl UA buffer three times and then with 100 μl DS buffer (50 mM triethylammonium bicarbonate at pH 8.5) twice. Finally, the protein suspensions were digested with 2 μg trypsin (Promega) in 40 μl DS buffer overnight at 37°C, and the resulting peptides were collected as a filtrate. The peptide content was estimated by UV light spectral density at 280 nm. A standard pool comprising a mixture of an equal amount of protein derived from all samples served as an internal control (IS). For labeling, each iTRAQ reagent was dissolved in 70 μl of ethanol, added to the respective peptide mixture, and then multiplexed and vacuum dried.

### Peptide fractionation with strong cation exchange (SCX) chromatography

iTRAQ-labeled peptides were fractionated by SCX chromatography using the AKTA Purifier system (GE Healthcare). The dried peptide mixture was dissolved in 2 mL buffer A (10 mM KH_2_PO_4_ in 25% acetonitrile, pH 3.0) and loaded onto a Polysulfoethyl column (4.6 × 100 mm, 5 μm, 200 Å, PolyLC Inc.). The peptides were eluted at a flow rate of 1 mL/min with a gradient of 0–10% buffer B (500 mM KCl, 10 mM KH_2_PO_4_ in 25% acetonitrile, pH 2.7) for 7 min, 10–20% buffer B for 10 min, 20–45% buffer B for 5 min, and 45–100% buffer B for 5 min. The eluates were monitored by absorbance at 214 nm and collected every 1 min. The collections were pooled in groups of 4 fractions and desalted separately on C18 Cartridges (Empore™ SPE Cartridges C18, standard density, bed I.D. 7 mm, volume 3 mL, Sigma). Each final fraction was dried in a vacuum concentrator and reconstituted in 40 μl of 0.1% (v/v) trifluoroacetic acid. All samples were stored at −80°C before the next analysis.

### Liquid chromatography (LC)-electrospray ionization (ESI) tandem mass spectrometry (MS) analysis by Q exactive

MS experiments were performed on a Q Exactive mass spectrometer that was coupled to a nanoflow HPLC instrument (Easy nLC, Thermo Fisher Scientific). The peptide mixture (5 μg) was loaded onto a C18-reversed phase column (Thermo Scientific Easy Column, 10 cm long, 75 μm diameter, 3 μm resin) in buffer A (0.1% formic acid) and separated in a linear gradient of buffer B (80% acetonitrile and 0.1% formic acid) at a flow rate of 250 nL/min over 140 min, controlled by IntelliFlow technology. MS data were acquired using a data-dependent “top10” method, dynamically choosing the most abundant precursor ions from the survey scan (300–1800 m/z) for HCD fragmentation. Determination of the target value was based on predictive Automatic Gain Control (pAGC). The dynamic exclusion duration was 60 s. Survey scans were acquired at a resolution of 70,000 at m/z 200, and the resolution for the HCD spectra was set to 17,500 at m/z 200. The normalized collision energy was 30 eV, and the underfill ratio, which specifies the minimum percentage of the target value likely to be reached at maximum fill time, was defined as 0.1%. The instrument was run with peptide recognition mode enabled.

### Sequence database search and data analysis

MS/MS spectra were searched using the MASCOT engine (Matrix Science, London, UK, version 2.2.) embedded into Proteome Discover 1.4 (Thermo Electron, San Jose, CA) against Uniprot_*A. baumannii*, Uniprot_*P. aeruginosa* and the corresponding decoy databases. Proteins were identified with the following parameters: Peptide mass tolerance = 20 ppm; MS/MS tolerance = 0.1 Da; Enzyme = trypsin; Missed cleavage = 2; Fixed modification: Carbamidomethyl (C), iTRAQ 4plex (K), iTRAQ 4plex (N-term); Variable modification: Oxidation (M). All reported data were based on 99% confidence intervals for protein identification as determined by a false discovery rate (FDR) ≤0.01 (Zheng et al., 2014).

The final ratios of proteins were normalized to the median average protein ratio of a mixture of equal volumes of differently labeled samples. Differentially expressed proteins were specified by a ratio of > ±1.2 and *p* < 0.05 (Cox and Mann, 2008). Screened proteins were loaded into Blast2GO (Version 2.7.0) for GO mapping and annotation (Ashburner et al., 2000; Gotz et al., 2008). These proteins were also mapped to KEGG pathways for further analysis (Kanehisa et al., 2012). Fisher's exact test was used to calculate *p*-values, and *p* < 0.05 indicated GO or KEGG pathways that were significantly enriched in differentially expressed proteins compared to the untreated group (Blüthgen et al., 2005).

## Results

### Cathelicidin-BF promptly lost its antibacterial activity in mouse serum

**Figure 1:**
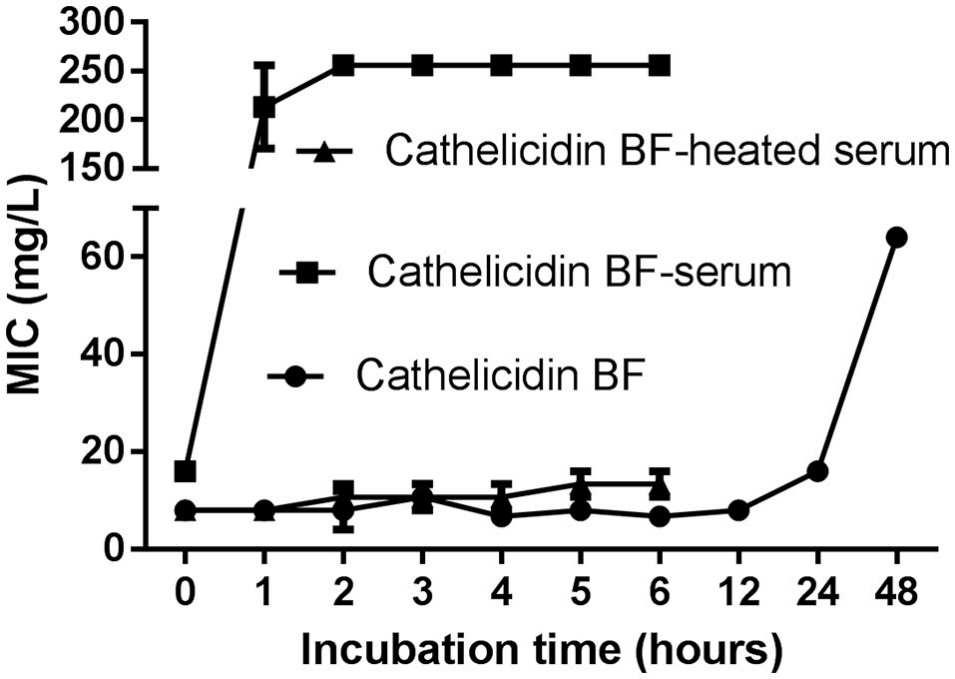
Serum stability of cathelicidin-BF. Cathelicidin-BF dissolved in sterile deionized water was mixed with mouse serum, heat-inactivated serum, or sterile deionized water at a volume ratio of 1:4 to reach a final concentration of 2 mg/mL. After incubation at 37°C, aliquots were taken at each time point, and the MIC against DH5α was defined as the lowest peptide concentration at which no microbial growth was observed visually after 18 h of incubation at 37°C. The stability of the MIC is an indicator of the stability of cathelicidin-BF.

Although cathelicidin-BF was stable after incubation in sterile deionized water at 37°C for nearly 24 h, reflected by the fact that the MIC against DH5α increased slightly from 8 mg/L to 16 mg/L, it almost completely lost its antibacterial activity after 1 h incubation in mouse serum, reflected by the fact that the MIC against DH5α increased to more than 128 mg/L (Figure 1). This process was so rapid that cathelicidin-BF lost half of its antibacterial activity immediately after being mixed with mouse serum, reflected by the fact that the MIC against DH5α was 8 mg/L after being mixed with water, while the MIC against DH5α was 16 mg/L after being mixed with serum at incubation time 0. When the serum was heat inactivated, the antimicrobial activity of cathelicidin-BF was relatively stable, as indicated by the slight increase in the MIC against DH5α from 8 mg/L at time 0 to approximately 13.3 mg/L after 6 h of incubation. These results are consistent with previous reports that linear AMPs are unstable *in vivo* because of endogenous mammalian proteases and proteases secreted by pathogens (Pasupuleti et al., 2009; Li et al., 2012).

### Cathelicidin-BF caused slight cytotoxicity to specific eukaryotic cells

**Figure 2:**
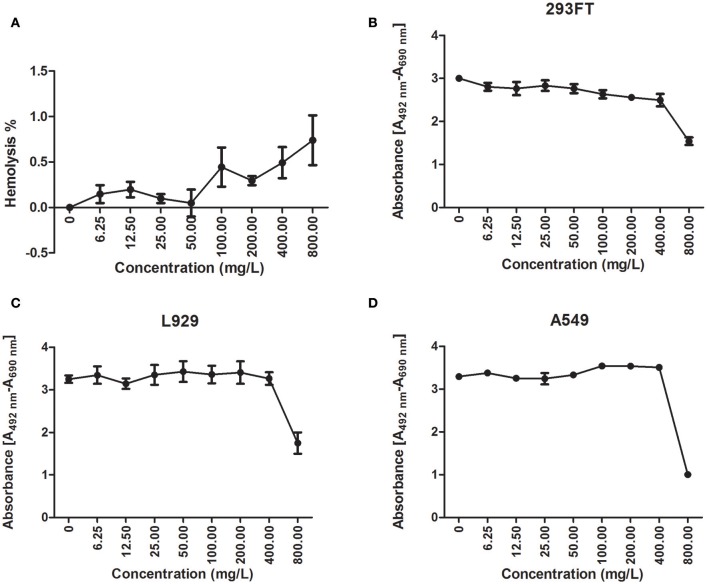
Cytotoxicity of cathelicidin-BF. Hemolysis and cell viability assays were conducted to test the cytotoxicity of cathelicidin-BF to mammalian cells. **(A)** For the hemolysis assay, cathelicidin-BF dissolved in 0.9% saline was added to mouse erythrocytes diluted in 0.9% saline and incubated at 37°C for 30 min. Supernatants were collected by centrifugation at 1,000 × g for 15 min and further diluted four times with 0.9% saline to test the absorbance at 540 nm. Using 1% Triton X-100 (v/v) to determine 100% hemolysis and 0.9% saline as the negative control, the hemolysis rate of cathelicidin-BF is expressed as [(Absorbance _sample_-Absorbance _control_)/(Absorbance_100%_-Absorbance _control_)]×100. The cell proliferation kit II (XTT) (Roche) was used to test the effects of cathelicidin-BF on the viability of **(B)** 293FT (human embryonic kidney cells), **(C)** L929 (mice fibroblast cell line) and **(D)** A549 (adenocarcinomic human alveolar basal epithelial cells) cells. Absorbance [A492 nm-A690 nm] was used to quantify viable cells.

Less than 1% hemolysis was observed when cathelicidin-BF was at a concentration of 800 mg/L (Figure 2A). Regarding other cells, cathelicidin-BF inhibited the proliferation of human embryonic kidney 293FT cells at concentrations >50 mg/L (Figure 2B) while leaving the murine fibroblast cell line L929 and adenocarcinomic human alveolar basal epithelial cell line A549 intact at 400 mg/L (Figures 2C, D). Our results are consistent with previous reports that a cathelicidin-BF mutant is toxic to Madin-Daby canine kidney (MDCK) cells at 20 μM (~72 mg/L) and may cause renal injury when applied systemically (Tian et al., 2013).

### Cathelicidin-BF could cross the bacterial membrane at low concentrations without detectable membrane disruption

**Figure 3:**
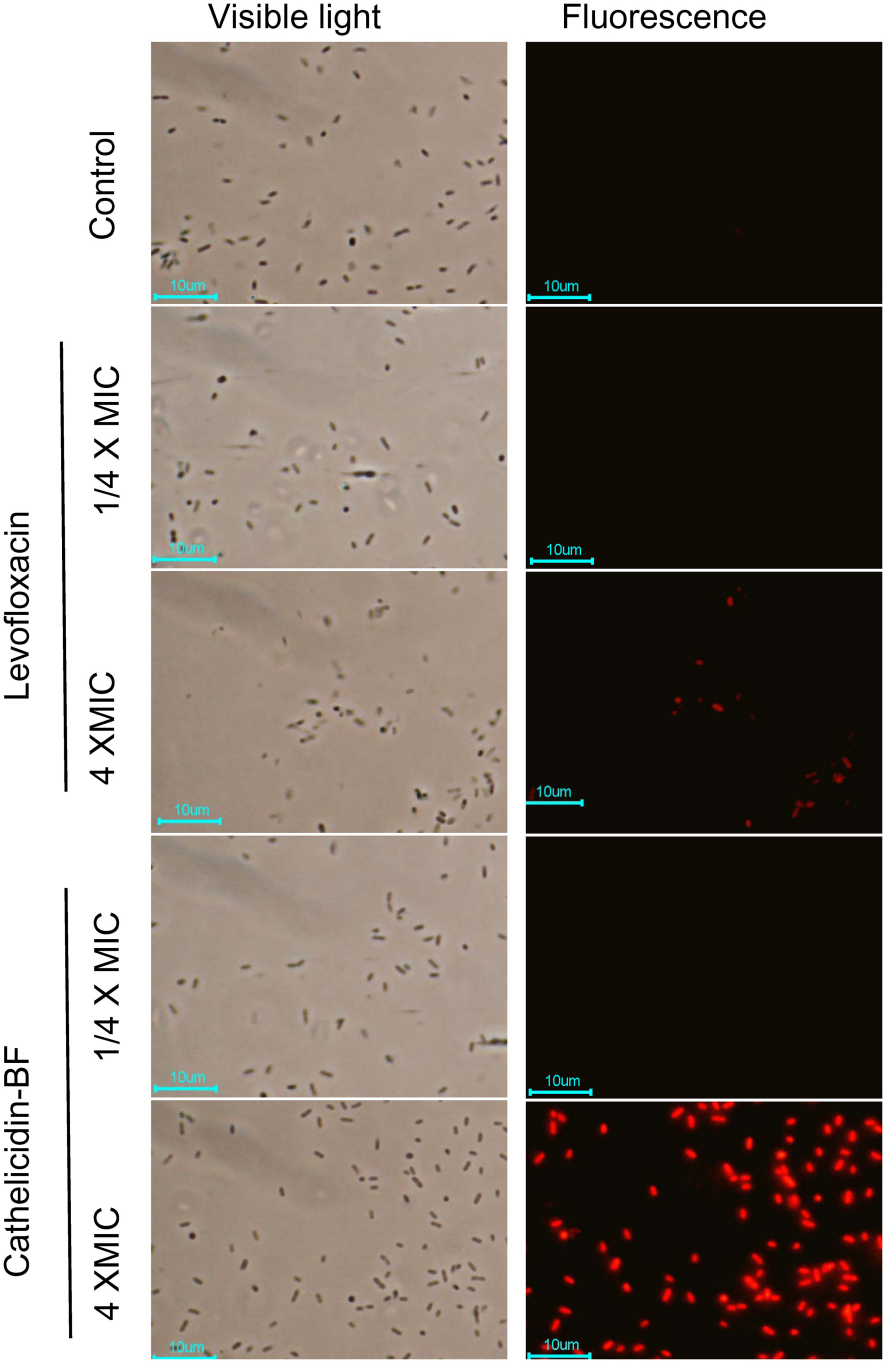
Bacterial plasma membrane permeabilization assay of cathelicidin-BF. Cathelicidin-BF and levofloxacin were incubated with MDR *P. aeruginosa* in Luria-Bertani medium at final concentrations of 4 × MIC (minimal inhibitory concentration) or ¼ × MIC. After incubation at 25°C for 1 h, bacteria were collected by centrifugation at 3,000 × g for 5 min and resuspended with PBS. The DNA-binding fluorescent dye PI was added to a final concentration of 10 μg/mL. After 30 min incubation at 25°C, the cells were washed 3 times with PBS and immediately imaged using a fluorescence microscope.

PI is a DNA-binding fluorescent dye that can penetrate broken membranes but not intact membranes. As shown in Figure 3, neither levofloxacin nor cathelicidin-BF at low concentrations (¼ × MIC) affects the integrity of bacterial membranes, as no red dyes were detected under these conditions. When the concentrations increased to 4 × MIC (a lethal concentration), both levofloxacin- and cathelicidin-BF-treated bacteria showed red fluorescence after incubation with PI, which indicated that the membranes of these bacteria were broken and that PI entered these cells and formed complexes with the DNA inside. Cathelicidin-treated membranes were noticeably more thoroughly disrupted than levofloxacin-treated ones. We are not sure whether these differences are due to the greater efficiency of cathelicidin-BF on bacteria or its potential actions on membranes or DNA compared with that of levofloxacin.

**Figure 4:**
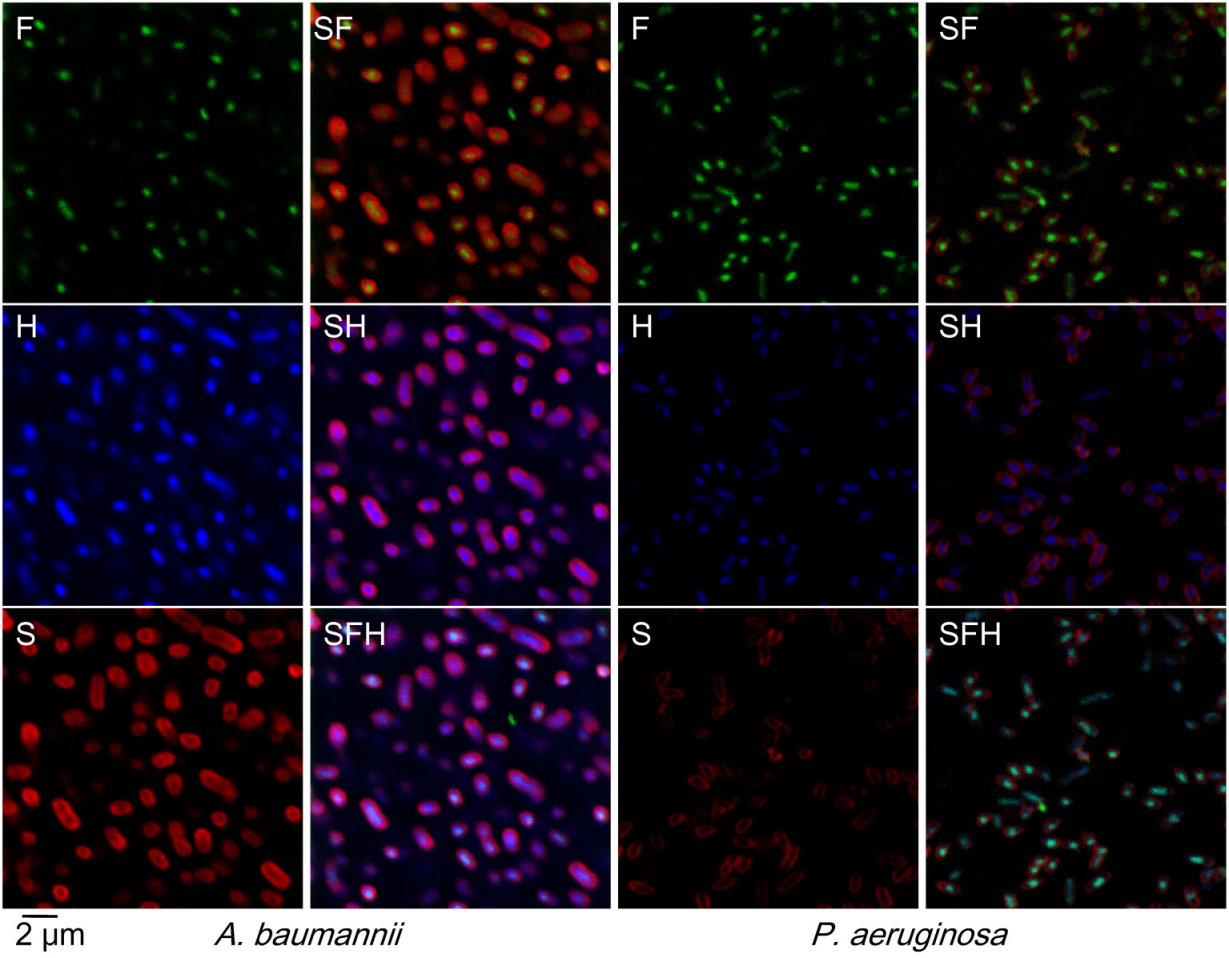
Localization of cathelicidin-BF in viable bacteria. N-terminus FITC (fluorescein isothiocyanate)-tagged cathelicidin-BF was added to final concentrations of ¼ × MIC for *P. aeruginosa* and *A. baumannii*. After incubation at 25°C for 1 h, bacteria were collected by centrifugation at 3,000 × g for 5 min, resuspended with PBS and then incubated with Hoechst at a final concentration of 20 μg/mL at room temperature for 20 min. After collection by centrifugation as described above, the bacteria were incubated with SynaptoRed in Hank's solution at a final concentration of 20 μg/mL on ice for 1 min. Microscopy was performed with excitation and emission wavelengths as follows: 488 nm and 530 nm for FITC (F, green color), 352 nm and 461 nm for Hoechst (H, blue color), 515 nm and 640 nm for SynaptoRed C2 (S, red color), respectively.

Indeed, cathelicidin-BF passed through bacterial membranes without disruption at low concentrations (Figure 4). Rather than localizing at membranes (stained with SynaptoRed C2 and shown in red in Figure 4, S) as we had previously expected according to the rupture thesis, FITC-tagged cathelicidin-BF (green in Figure 4, F) localized at nuclear regions, which is indicated by its co-localization with DNA (stained with Hoechst and shown in blue in Figure 4, H). Notably, FITC-tagged cathelicidin-BF seems to be more concentrated compared with the distribution of DNA at the nuclear region, suggesting that cathelicidin-BF may act on specific regions of the nucleoid.

### Go suggested potential intracellular targets of cathelicidin-BF

The functional interpretation of differentially expressed proteins (Tables S1–S3) was enriched via GO analysis. Interestingly, cathelicidin-BF- and levofloxacin-treated *P. aeruginosa* shared many GO categories concerning the transcription process (Table 1). These similarities also existed in cathelicidin-BF-treated *A. baumannii*, with shared processes, including core RNA polymerase binding (Table 2). Notably, both cathelicidin-BF- and levofloxacin-treated *P. aeruginosa* have their own specific GO categories. For example, GO categories concerning nucleic acids from cathelicidin-BF-treated *P. aeruginosa* mainly involve RNA (i.e., tRNA 3′-terminal CCA addition, RNA repair, etc.,), while GO categories concerning nucleic acids from levofloxacin-treated *P. aeruginosa* primarily involved DNA (i.e., primosome complex, replisome, double-strand break repair, etc.,) (Table 3). These results are consistent with the mechanisms of levofloxacin-mediated killing of bacteria (inhibition of two type II topoisomerases, namely, DNA gyrase and topoisomerase IV, which are involved in DNA separation and supercoiling, respectively) and implied that although both molecules target the nucleoid, cathelicidin-BF may have different mechanisms from levofloxacin (Drlica and Zhao, 1997; Ferrandiz and de la Campa, 2014).

**Table 1:** GO categories shared between cathelicidin-BF- and levofloxacin-treated *P. aeruginosa* (*p* < 0.05).

**GO ID**	**GO category**	***p*-value**
GO:0016989	Sigma factor antagonist activity	0.003
		0.005
GO:0000989	Transcription factor activity, transcription factor binding	0.003
		0.005
GO:0006355	Regulation of transcription, DNA-templated	0.005
		0.014
GO:2001141	Regulation of RNA biosynthetic process	0.012
		0.032
GO:1903506	Regulation of nucleic acid-templated transcription	0.012
		0.032
GO:0010468	Regulation of gene expression	0.015
		0.018
GO:2000112	Regulation of cellular macromolecule biosynthetic process	0.016
		0.019
GO:0010556	Regulation of macromolecule biosynthetic process	0.016
		0.019
GO:0031326	Regulation of cellular biosynthetic process	0.017
		0.020
GO:0006351	Transcription, DNA-templated	0.017
		0.041
GO:0097659	nucleic acid-templated transcription	0.017
		0.042
GO:0009889	Regulation of biosynthetic process	0.019
		0.023
GO:0051252	Regulation of RNA metabolic process	0.020
		0.047
GO:0032774	RNA biosynthetic process	0.020
		0.020
GO:0019219	regulation of nucleobase-containing compound metabolic process	0.023
		0.023
GO:0000988	Transcription factor activity, protein binding	0.025
		0.036
GO:0016070	RNA metabolic process	0.025
		0.009
GO:0051171	Regulation of nitrogen compound metabolic process	0.027
		0.032
GO:0060255	Regulation of macromolecule metabolic process	0.028
		0.033
GO:0080090	Regulation of primary metabolic process	0.029
		0.034
GO:0031323	Regulation of cellular metabolic process	0.031
		0.037
GO:0072509	Divalent inorganic cation transmembrane transporter activity	0.033
		0.041
GO:0015197	Peptide transporter activity	0.033
		0.041
GO:0015095	Magnesium ion transmembrane transporter activity	0.033
		0.041
GO:0015693	Magnesium ion transport	0.033
		0.041
GO:0019222	Regulation of metabolic process	0.036
		0.043
GO:0090304	Nucleic acid metabolic process	0.039
		0.019

*The p-values of levofloxacin-treated P. aeruginosa are shaded, whereas those of cathelicidin-BF-treated cells are not*.

**Table 2:** GO categories for cathelicidin-BF-treated *A. baumannii*.

**GO ID**	**GO category**	***p*-value**
GO:0016987	Sigma factor activity	0.008
GO:0006352	DNA-templated transcription, initiation	0.008
GO:0000996	Core DNA-dependent RNA polymerase binding promoter specificity activity	0.008
GO:0000990	Transcription factor activity, core RNA polymerase binding	0.008
GO:0000988	Transcription factor activity, protein binding	0.008

*Only GO categories with p < 0.01 are shown*.

**Table 3:** Differences in GO categories related to nucleic acids between cathelicidin-BF- and levofloxacin-treated *P. aeruginosa* (*p* < 0.05).

**GO ID**	**GO category**	***p*-value**
GO:0010629	Negative regulation of gene expression	0.031077
GO:0001680	tRNA 3′-terminal CCA addition	0.033263
GO:0017148	Negative regulation of translation	0.033263
GO:0042245	RNA repair	0.033263
GO:0001071	Nucleic acid binding transcription factor activity	0.044257
GO:0003700	transcription factor activity, sequence-specific DNA binding	0.044257
GO:0005667	Transcription factor complex	0.044257
GO:2000104	Negative regulation of DNA-dependent DNA replication	0.041047
GO:1990077	Primosome complex	0.041047
GO:0006302	double-strand break repair	0.041047
GO:0090329	Regulation of DNA-dependent DNA replication	0.041047
GO:0006269	DNA replication, synthesis of RNA primer	0.041047
GO:0030894	Replisome	0.041047
GO:0030174	Regulation of DNA-dependent DNA replication initiation	0.041047
GO:0032297	Negative regulation of DNA-dependent DNA replication initiation	0.041047

*The GO categories enriched in levofloxacin-treated P. aeruginosa are shaded, whereas those enriched in cathelicidin-BF-treated cells are not*.

### KEGG analysis confirmed intracellular targets

KEGG pathway maps represent experimental knowledge on metabolism and various other functions of the cell and organism. Localization of differentially expressed proteins in these pathways may offer hints to how drugs work by interfering with key metabolic activities. As seen in Table 4, differentially expressed proteins from *P. aeruginosa* after cathelicidin-BF or levofloxacin treatment shared many pathways, including amino acid synthesis and pyrimidine and purine metabolism. Some of these pathways were further confirmed by their existence in cathelicidin-BF treated *A. baumannii* (KEGG categories with MapIDs are underlined in Table 4). Notably, while some of these pathways were not “enriched” (*p* > 0.05), they share some differently expressed proteins with other enriched pathways. For example, proteins Q9HVA1 and Q9I3S7 are shared in six pathways, although they are considered enriched in only 4 of them. Q9HVA1 is the acetolactate synthase isozyme III small subunit with functions in branched-chain amino acid biosynthesis and metabolism of cofactors and vitamins. It is overexpressed in both cathelicidin-BF- and levofloxacin-treated *P. aeruginosa*. Q9I3S7 is a likely decarboxylase that may interact selectively and non-covalently with thiamine pyrophosphate (the diphosphoric ester of thiamine, TPP). It is overexpressed in levofloxacin-treated *P. aeruginosa* but downregulated in cathelicidin-BF-treated *P. aeruginosa*. A similar phenomenon occurred with Q9HUU8, the urease subunit gamma that is involved in arginine biosynthesis and purine metabolism.

**Table 4:** KEGG categories shared between cathelicidin-BF- and levofloxacin-treated *P. aeruginosa*.

**Map ID**	**Map name**	**Genes shared**	***p*-value**
ko02010	ABC transporters	Q9HVR6 Q9I33L L9 Q9I5T5	0.025
			0.046
ko00920	Sulfur metabolism	Q9I33L L9	
			
ko00660	C5-branched dibasic acid metabolism	Q9HVA1 Q9I3S7	0.025
			0.003
ko01230	Biosynthesis of amino acids	Q9HVA1 Q9I3S7	
			
ko00770	Pantothenate and CoA biosynthesis	Q9HVA1 Q9I3S7	
			0.013
ko00220	Arginine biosynthesis	Q9HUU8	
			
ko00650	Butanoate metabolism	Q9HVA1 Q9I3S7	
			
ko02020	Two-component system		
			
ko00791	Atrazine degradation	Q9HUU8	
			
ko01210	2-oxocarboxylic acid metabolism	Q9HVA1 Q9I3S7	
			0.016
ko00290	Valine, leucine and isoleucine biosynthesis	Q9HVA1 Q9I3S7	
			0.001
ko00240	Pyrimidine metabolism		
			
ko00230	Purine metabolism	Q9HUU8	
			

*The p-values of levofloxacin-treated P. aeruginosa are shaded, whereas those of cathelicidin-BF-treated cells are not. Underlined map IDs are also shared with cathelicidin-BF-treated A. baumannii*.

Unique pathways were identified for each treatment. Q9I5V3, the multifunctional CCA protein, is downregulated in cathelicidin-BF-treated *P. aeruginosa* and enriched in the RNA transport pathway. Ubiquinone and other terpenoid-quinone biosynthesis pathways were enriched in levofloxacin-treated *P. aeruginosa*. Q9I298, a putative 3-methylglutaconyl-ConA hydratase, and Q9HTV3, a 3-octaprenyl-4-hydroxybenzoate carboxy-lyase, were downregulated in this pathway. Notably, Q9I5V3 was downregulated in levofloxacin-treated *P. aeruginosa*, and both Q9I298 and Q9HTV3 were downregulated in cathelicidin-BF-treated *P. aeruginosa* but they are not categorized as “differentially expressed proteins” according to the criteria set in the method section.

Interestingly, we found one protein, Q9I523, a nucleoside-triphosphate diphosphatase that is involved in both purine and pyrimidine metabolism, that was downregulated in cathelicidin-BF-treated *P. aeruginosa*. This protein was reported to interact with Era, an essential GTPase identified in various bacteria and some eukaryotes (Zhang and Inouye, 2002). Recently, a study reported that AMPs can kill bacteria by inhibiting *E. coli* ATP synthase (Azim et al., 2016). Considering that Q9I523 could hydrolyze all eight of the canonical ribo- and deoxynucleoside triphosphates to their respective monophosphates and PP (i), downregulation of this “energy switch” may play an important role in regulating the energy supply of a bacterial or tumor cell.

## Discussion

AMPs are effective for some of the clinical superbugs, including *P. aeruginosa* and *A. baumannii*, but they have not been able to be administered systemically, mainly because of their poor stability *in vivo* and possible toxic effects, such as hemolysis or immunoregulation. Although AMPs were discovered several decades ago, the mechanism by which they kill bacteria is still controversial. In recent years, the membrane rupture thesis has been challenged by the fact that some AMPs can kill not only bacteria but also viruses, fungi, protozoa, parasites and cancer cells. In addition, more and more AMPs have been reported to have intracellular targets (Shah et al., 2016).

Cathelicidin-BF is a typical cationic, amphiphilic and α-helical AMP that has powerful effects on MDR clinical superbugs but cannot be applied systemically due to its sensitivity to proteases *in vivo*. In this report, we demonstrated that the disruption of the bacterial membrane at high concentrations of cathelicidin-BF was the result of bacteria death, as is the case for conventional antibiotics at high concentrations (Figure 3). In fact, lower concentrations of cathelicidin-BF did not cause bacterial membrane damage but could cross the membrane and aggregate at nucleoid regions (Figure 4). Therefore, we used levofloxacin for comparison in further analysis because it kills bacteria by interfering with DNA separation and supercoiling, which also occurs at nucleoid regions. Comparative proteomics showed that cathelicidin-BF may affect transcription in a similar way as levofloxacin (Tables 1, 2), which is consistent with its localization in the nucleoid regions after incubation. However, cathelicidin-BF tends to affect RNA synthesis, while levofloxacin tends to affect DNA replication (Table 3), which implies that these molecules may have different mechanisms of killing bacteria. These similarities and differences could also be seen from the KEGG analysis. While the mechanisms shared genes involved in the biosynthesis of amino acids and purine metabolism, cathelicidin-BF specifically affects pathways involving RNA transport, and levofloxacin has unique effects on quinone biosynthesis (Table 4). Moreover, we found one downregulated nucleoside-triphosphate diphosphatase in cathelicidin-BF-treated *P. aeruginosa*, which offered hints that AMPs may kill bacteria by controlling the energy supply.

Although our study provided evidence that cathelicidin-BF may act on intracellular targets instead of membranes to kill bacteria, several questions still need to be answered: (1) What are the specific targets of cathelicidin-BF when it kills the bacteria? We are not sure whether it kills the bacteria by affecting key enzymes involved in translation, as levofloxacin does, or considering the absence of nuclear membranes in bacteria, whether it simply binds to the negatively charged nucleic acids after crossing plasma membranes because of its cationic characteristic and subsequently interrupts the functions of these nucleic acids. (2) If cathelicidin-BF has protein targets, does it target specific multifunctional enzymes or multiple key enzymes to kill bacteria? We have noticed that cathelicidin-BF interferes with multiple metabolic processes of bacteria, including amino acid synthesis, metabolism of cofactors and vitamins, and metabolism of purines and energy supply. Further steps are still needed to confirm the contributions of the bactericidal effects of these differentially expressed proteins. (3) What is the contribution of the plasma membrane-crossing activity of cathelicidin-BF in killing bacteria? Considering that cathelicidin-BF may have similar targets as levofloxacin but that *A. baumannii* 1408 and *P. aeruginosa* 1409 have gained resistance to levofloxacin (possibly by efflux pumps), understanding how cathelicidin-BF crosses the bacteria plasma membranes (e.g., receptors, biophysical characteristics, etc.,) may also contribute to further discoveries of novel antibiotics targeting superbugs.

## Author contributions

Conceived and designed the experiments: CL; performed the experiments: CL, BS, and JQ; analyzed the data: CL and BS; contributed reagents/materials/analysis tools: BS; contributed to the writing of the manuscript: CL and YM.

### Conflict of interest statement

The authors declare that the research was conducted in the absence of any commercial or financial relationships that could be construed as a potential conflict of interest.

## Supplementary material

Table S1 Peptide identification.

Table S2 Protein identification.

Table S3 Quantivative and differential analysis of proteins.
